# Books Reviewed

**Published:** 1863-12

**Authors:** 


### BOOKS REVIEWED.

*The Principles and Practice of Ophthalmic Medicine and Surgery.* By T. Wharton Jones, F. R. S., *Professor of Ophthalmic Medicine and Surgery in University College, London; Ophthalmic Surgeon to the Hospital, etc., with one hundred and seventeen Illustrations. Third and Revised American Edition, with additions from the Second London Edition. Philadelphia:* Blanchard & Lea, 1863.

There is no department of Medicine or Surgery which has undergone more changes and been enriched with greater improvements during the last ten years than that of Ophthalmic Medicine and Surgery. Our knowledge of the pathology of diseases of the eye has been greatly increased, particularly of the deeply seated structures, while at the same time our therapeutics has received thorough revision and improvement, and operative procedures have been instituted by which many ophthalmic affections can be relieved. This field of practice has been cultivated with great ability, and results have been obtained which do honor to the laborers who have been chiefly instrumental in the discovery of new truths, and application of improved means of relief.

From the examination of this book, which we have been able to make, we are satisfied that it is fully up to the present advanced state of ophthalmic knowledge, and that nothing practically useful has been omitted which could be possibly embraced in a work of 500 pages, which is designed for the guide of the practitioner, available at the bed-side of the patient, and in the operating room.

The instruction given upon the uses of the ophthalmoscope, and the illustrations of the diseases which this instrument is capable of showing constitute a valuable chapter.

Within the last few years the operation of iridictomy, or excising a segment of the iris for the relief of glaucoma, or to relieve intra occular pressure has attracted a great deal of attention, and much has been written upon the subject. It may be interesting to quote a few remarks upon this subject since the theory, operation and probable results, are condensed in few words:

"All the leading features of glaucoma are to be attributed—according to the most recent authorities—to excessive tension of the eyeball from a superabundance of fluid within it, which distends the vitreous humor. This fluid—serum—is derived from the choroid, so that glaucoma might be considered a serous choroiditis.

It is held, therefore, that the loss of vision in the condition of early glaucoma is not the result of any change primarily occurring in the retina, but of a pressure of the vessels, and that by the removal of this pressure, the retina will regain its power, just as by compression within the head the brain might lose its functions for a time, and regain them when the pressure was removed.

In accordance with these views, Graefe excised a portion of the iris in a particular way, and stated that there was almost from the first, a diminution of pressure, followed by an abatement of the symptoms, and an improvement of the vision, even when under great pressure, it had been abolished for a time.

This operation of Graefe, called iridictomy, consists in excising a segment of the iris in its whole breadth, from the pupillary margin outwards to its insertion through an opening of corresponding size, made at the extreme edge of the anterior chamber. By the removal of the iris in this manner, the pupil is at once enlarged up to the corneal incision, which forms, as it were, the base of a colobonia iridis, and the edge of the lens, with the suspensory ligament, stretching in front of the vitreous humor, and the ciliary processes is exposed to view. The little blood which oozes into the anterior chamber from the cut edges or surface of the iris should be at once pressed out or removed with a scoop. The after-treatment is very simple. A light compress may be applied for a short time, as a precaution against hæmmorrhage. This may be replaced after an hour or two, by a piece of wet rag. The room should be shaded. At first, the aqueous humor trickles away, but the corneal wound soon heals, and the anterior chamber fills again. The hardness of the eye ball is at once lessened, and a natural tension is gradually attained; the pain abates, and soon altogether disappears. As regards vision, when the proper cases are selected for the operation, it is claimed by many surgeons, to be completely restored.

We believe, ourselves, that the operation of iridictomy is of no use in chronic glaucoma, while in acute glaucoma, good effects do result from the operation; notwithstanding this, we are persuaded that the excision of the iris is a proceeding unnecessarily superadded to a means long known as calculated to give relief, and to which alone the benefit obtained is to be attributed, namely: the removal of the tension by evacuation of the superabundant fluid in the eye."

Division of the ciliary muscle, as proposed by Mr. Hancock, is also noticed in this connection, but we have not space to devote to this subject, and must close our notice of this work, which we regard as especially valuable to the general practitioner. The numerous illustrations which it contains are indispensable in assisting to form correct diagnosis, or in illustrating the manner of making operations. The book, as a guide and reference, is invaluable, and will, we have no doubt, in its present improved edition, more than ever, receive confidence, and be regarded as standard authority, upon the subjects it treats.

---

*Mental Hygiene*—By J. Ray, M. D. *Boston:* Ticknor & Fields, 1863.

The work before us is divided into five chapters, on Mental Hygiene, as affected by Cerebral Conditions—by Physical Influences—by Mental Conditions and Influences—by the Practices of the Times, and by Tendency to Disease.

The first chapter discusses the relations of the mind to the brain. He says:

"As the music is in the player, not in the instrument he uses, so is the brain the material organ by which the mind is enabled to exercise its powers. On the other hand, among men whose views on philosophical subjects are determined more by the testimony of sense than any subtle deductions of reason, there are some who regard the mind as entirely a function of the brain. Without the brain there is, and there can be, no mind. This question, as already hinted, derives its importance chiefly from the theological bearings. If the mind is an original, independent principle, having only an incidental connection with the body, then, it is supposed, it may, and indeed must exist after the dissolution of the body. But, if it is merely a phenomenon resulting from the play of organic elements, it must necessarily perish with the organism from which it sprung. It is not quite certain that either of these views will warrant the inference that is drawn from it. Although we may admit the independent existence of the mind, there must be other reasons, I apprehend, for believing it to be immortal; and, though we admit that the mind is a product of vital movements, it does not necessarily follow that there can be no conscious existence after the component parts of the animal mechanism are dispersed. We are not to measure the resources of Almighty Power by our own feeble conceptions, nor to suppose that a fact is impossible merely because some of its conditions are beyond our comprehension."

Dr. Ray does not believe in the indefinite progressive development of any special traits, and thinks the world will look in vain for any higher types of mind than it has already seen, yet he does not underrate the value of studying the laws of hereditary transmission, and following their teachings. He remarks as follows:

"Now, what we seek for as the proper result and aim of mental cultivation is, not a particular endowment that may be transmitted from one generation to another, but a large range of capacity, great facility of achievement, and great power of endurance. That these qualities may be rendered permanent by a faithful compliance with the laws of breeding, there can scarcely be a doubt; but this, it must be observed, is something very far short of indefinite development. We have no reason to suppose that, by any possible scheme of training and breeding, finer specimens of the race can be obtained than Pericles and Alcibiades; but we are warranted in believing that by this means individuals of distinguished general excellence would be far more common. If it be true, then, that, in the various stages of its progress, the mind, like the body, is under the government of inflexible laws. it follows that these laws should be thoroughly understood, in order to obtain the highest possible degree of mental efficiency. To show exactly what they are, to exhibit the consequences that flow from obeying or disobeying them, is the essential object of mental Hygiene, which may be defined as the art of preserving the health of the mind against all the incidents and influences calculated to deteriorate its qualities, impair its energies, or derange its movements. The management of the bodily powers in regard to exercise, rest, food, clothing and climate; the laws of breeding, the government of the passions, the sympathy with current emotions and opinions, the discipline of the intellect—all come within the province of mental hygiene."

Upon the subject of intermarriage between blood relations, he says:

"A not unfrequent cause of mental deterioration, is the intermarriage of blood relations. The great physiological law, that like produces like, depends upon this condition, that the parents shall not be nearly allied by blood. In the domestic animals, neglect of this condition is soon followed by deterioration, and if continued through several generations, the original good qualities of the breed disappear altogether. In man this effect is less obvious, parties often escaping any apparent penalty, even when the law is violated in two successive generations. But it is common enough and severe enough to render infractions of the law fearfully hazardous. Its existence has been denied on the strength of some limited statistics, but the stern facts on the subject are too numerous to be accidental, and it must be our own fault if we do not heed the lesson which they teach. Because the physical qualities of the parents are occasionally too prominent and too well established to be materially injured by a single infringement of the law, and the first impression is not enforced and reduplicated by repetitions of the infringement, men are disposed to believe that they have committed no transgression!

"Within a few years past, the physiological effects upon the offspring or marriages in consanguinity, have been carefully investigated by Devay. Perrin, Menière, and others, in France, and Bemiss and Howe in this country. These inquiries show, among these effects, an extraordinary proportion of disease and imperfection in the shape of insanity, idiocy, epilepsy, blindness, deaf-mutism and sterility. From 24 to 30 per cent. of all the pupils in the institutions of France for deaf mutes are the offspring of such marriages, and many of them left a deaf mute brother or sister at home. Dr. Howe collected the statistics of seventeen marriages in consanguinity, from which it appears that of the ninety-five children which proceeded from them, forty-four were idiots, twelve scrofulous and delicate, one deaf, and one a dwarf. Dr. Bemiss has collected the results of eight hundred and thirty-three consanguineous marriages, reported by himself and others, from which proceeded thirty nine hundred and forty-two children. Of these, one hundred and forty-five were deaf mutes, eighty-five blind, three hundred and eight idiotic, thirty-eight insane, sixty epileptic, three hundred scrofulous, ninety-eight deformed, and one hundred defective in one way or another."

This work is written in fine style, and good taste, and is designed for popular use, and contains suggestions quite as much for the benefit of the masses as for the professional reader. We hope it may receive general attention, for it tells us many things, some for us to do, and some to leave undone, that we may enjoy the highest degree of mental health.

---

